# Robot‐assisted versus laparoscopic partial nephrectomy for anatomically complex T1b renal tumors with a RENAL nephrometry score ≥7: A propensity score‐based analysis

**DOI:** 10.1002/cam4.2749

**Published:** 2019-12-02

**Authors:** Wen Deng, Junhua Li, Xiaoqiang Liu, Luyao Chen, Weipeng Liu, Xiaochen Zhou, Jingyu Zhu, Bin Fu, Gongxian Wang

**Affiliations:** ^1^ Department of Urology The First Affiliated Hospital of Nanchang University Nanchang Jiangxi China; ^2^ Jiangxi Institute of Urology Nanchang Jiangxi China; ^3^ Department of Urology Third Hospital of Hangzhou Hangzhou Zhejiang China

**Keywords:** laparoscopy, partial nephrectomy, renal tumor, robotics, T1b

## Abstract

**Objectives:**

To present the perioperative, functional, and oncological outcomes of robot‐assisted partial nephrectomy (RPN) compared with laparoscopic partial nephrectomy (LPN) for anatomically complex T1b renal tumors with RENAL nephrometry scores ≥7.

**Patients and methods:**

One hundred and seventy patients, during the study period, were retrospectively reviewed in our analysis according to inclusion criteria. Propensity score matching (PSM) (1:1) method was applied to impose restrictions on the potential baseline confounders. The comparisons of perioperative and functional outcomes between the RPN and LPN groups were conducted and analyzed after PSM, Kaplan‐Meier analyses were performed to assess the differences about oncological outcomes between the two groups before and after PSM.

**Results:**

One hundred and nine and 61 T1b renal tumors with RENAL scores ≥7 were identified in the LPN and RPN groups, respectively. All significant differences in baseline characteristics disappeared after PSM. Except for 3 patients missing an appropriate pair, all the patients in the RPN group were successfully matched to 58 patients in the LPN group in a 1:1 ratio. Within the matched cohort, the RPN group was related to a significantly shorter mean operating time (OT) (*P* = .040), shorter mean warm ischemia time (WIT) (*P* = .023), and shorter median postoperative hospital stay (*P* = .023). The possibilities of surgical conversion, postoperative complication, and positive surgical margin were similar in the LPN and RPN groups. And there was also no significant difference in the pathological, renal functional, and oncological outcomes between the two series.

**Conclusions:**

For patients with anatomically complex T1b renal tumors with a RENAL nephrometry score ≥7, RPN had an advantage over LPN in reducing OT, WIT, and postoperative hospital stay length without increasing the risk of complications and weakening the oncological control, while the two surgical methods were similar in renal functional preservation.

## Graphical abstract

For patients with anatomically complex T1b renal tumors with a RENAL nephrometry score ≥7, RPN had an advantage over LPN in reducing the operating time, warm ischemia time and postoperative hospital stay length without increasing the risk of complications and weakening the oncological control, while the two surgical methods were similar in renal functional preservation.

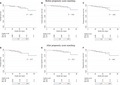

## 1 INTRODUCTION

Depending on the advantage in better renal functional preservation without compromising the oncological control, partial nephrectomy (PN) has been strongly recommended as the standard surgical method for T1a or even T1b renal tumors when technically feasible.^1^ The better preservation of renal function after PN than that after radical nephrectomy (RN) was relative to the reduction of overall mortalities and lower possibility of metabolic or cardiovascular disorders.^1^, ^2^, ^3^, ^4^ Minimally invasive PN (MIPN), namely, laparoscopic PN (LPN) and robot‐assisted PN (RPN), has received increasingly extensive acceptance for selected renal tumors with the improvement in techniques, equipment, and operator skills.^5^, ^6^ Compared with open PN, MIPN had the superiority in relieving postoperative pain and shortening hospital stay length whilst achieving similar cancer control.^7^, ^8^

The main advantages consisting of the three‐dimensional (3D) magnified vision of the surgical field, improved dexterity, and higher precision in the surgical procedure made RPN a great evolution of LPN.^7^, ^9^ Many studies had demonstrated the safety and efficiency of RPN for complex renal tumors,^9^, ^10^, ^11^, ^12^, ^13^ but most tumors in all these studies were in small size (≤4 cm) and these evidences were insufficient about oncological outcomes. No study concentrated on comparing LPN and RPN for anatomically complex T1b renal tumors with a RENAL nephrometry score ≥7 before, which was of great importance and remained debated.^14^ In the current study, we present the first comparison of the perioperative, functional, and oncological outcomes between LPN and RPN for anatomically complex T1b renal tumors with a RENAL nephrometry score ≥7 before and after the propensity score matching (PSM).

## 2 PATIENTS AND METHODS

The obtainment of baseline demographics and clinical information was retrospective by scrutinizing our prospectively maintained database after acquiring the approval of the institutional review board and ethics committee. Tumor complexity was assessed through reviewing the radiological imaging of renal tumor by the same highly experienced surgeon (Luyao Chen) on the basis of RENAL nephrometry score.^15^ One hundred and seventy patients with complex T1b renal tumors were extracted and included in our final analysis between June 2010 and December 2017 according to the following inclusion criterion: (a) patients with clinical T1b renal tumor, without multiple or bilateral neoplasm and metastatic diseases; (b) renal tumors with a RENAL nephrometry score ≥7; (c) patients underwent RPN or LPN for renal tumors. Only when the patients were eligible for all these criteria simultaneously were they included. Otherwise, the one would be excluded from this analysis. 170 patients were classified into the LPN (n = 109) and RPN (n = 61) groups according to the surgical type. All LPN procedures were completed by four extensively experienced surgeons during the whole study period, while the RPN procedures were performed by the same surgeons since January 2015. Transperitoneal or retroperitoneal approach was applied depending on the preference of these surgeons. The treatment assignment in our retrospectively designed study was usually at the discretion of the highly experienced surgeons according to tumor and patient characteristics. The existing studies had presented the detailed procedures of LPN and RPN.^16^, ^17^

Baseline demographics and clinical features (age, gender, diabetes mellitus, hypertension, American Society of Anesthesiologists [ASA] score, RENAL score, preoperative creatinine, estimated glomerular filtration rates [eGFR], chronic kidney disease [CKD] stage, and hemoglobin) were extracted from the database. Tumor features included tumor size and tumor laterality by evaluating the computed tomography. Perioperative results included surgical approach, operating time (OT), estimated blood loss (EBL), warm ischemia time (WIT), transfusion, conversion (to radical surgery or open surgery), positive surgical margin (PM), postoperative hospital stay, postoperative complication assessed according to Clavien‐Dindo classification.^18^ Pathological outcomes included pathologic stage, fuhrman grade and histologic subtype. Renal functional outcomes were evaluated by eGFR within postoperative 1 week, last follow‐up eGFR, eGFR decrease in baseline and the occurrence of de novo CKD at the last follow‐up. The follow‐up arrangements were regular for each patient during the postoperative period. Overall survival (OS), cancer‐specific survival (CSS) and progression‐free survival (PFS) were defined as the intervals from the date of surgery to that of death caused by anything, death caused by renal tumor and recurrence or metastasis, respectively.

PSM method was applied to eliminate any significant difference in preoperative clinical characteristics. Nonparsimonious and multivariate logistic regression was utilized to calculate the propensity scores on the basis of all preoperative features. Considering the intra‐ and postoperative results could not impact the surgical option, we excluded the intra‐ and postoperative outcomes out of the PSM process. Except for 3 patients who lacked a suitable pair, 58 patients in the RPN group were perfectly matched to 58 patients in the LPN group in a 1:1 ratio according to the nearest neighbor matching method.

All continuous variables in a normal distribution were presented as mean and standard deviation (SD) and compared utilizing the independent *t* test, while the non‐normally distributed ones were described as median and interquartile range (IQR) and analyzed using the Wilcoxon‐rank sum test. Categorical variables were compared employing the Pearson chi‐squared or Fishers' exact test. The Kaplan‐Meier method was performed before and after PSM to assess the survival outcomes of the patients with malignant tumors using log‐rank test. The survival outcomes of the LPN and RPN groups after PSM were compared further according to different surgical approaches. The IBM SPSS version 22 statistical package (SPSS Inc) was employed to conduct all statistical analyses except for Kaplan‐Meier analyses, which were performed using Stata SE 12.0 software. Statistical significance was set as a two‐side *P* < .05.

## 3 RESULTS

One hundred and nine and 61 patients were included in the LPN and RPN groups, respectively, in accordance to the inclusion criteria. Table 1 has described the preoperative characteristics in detail. Before the PSM, the rate of solitary kidney in the LPN group was significantly higher than that in the RPN group (12.8% vs 3.3%, *P* = .040). The LPN group was in relation to a significantly higher proportion of patients with preoperative CKD (11.0% vs 1.6%, *P* = .027). Meanwhile, patients in the LPN group had a lower mean preoperative hemoglobin level (116.3 vs 123.0 g/L, *P* = .025). No statistically significant differences in other variables were found between the LPN and RPN groups. After the PSM, 58 patients in the RPN group were successfully matched to 58 patients in the LPN group with an enhanced balance for all preoperative characteristics, and the statistically significant differences in the rates of solitary kidney and preoperative CKD and the mean preoperative hemoglobin level have disappeared between the two groups (Table 1).

**Table 1:** Preoperative characteristics by surgery type before and after propensity score matching

Variable	Before propensity score matching			After propensity score matching		
	LPN (n = 109)	RPN (n = 61)	*P* value	LPN (n = 58)	RPN (n = 58)	*P* value
Age, y, mean (SD)	50.6 (9.5)	52.8 (10.3)	.168	50.6 (9.8)	52.0 (10.0)	.449
Gender (male), n (%)	63 (57.8)	31 (50.8)	.380	32 (55.2)	31 (53.4)	.852
Diabetes mellitus (yes), n (%)	13 (11.9)	7 (11.5)	.930	6 (10.3)	7 (12.1)	.769
Hypertension (yes), n (%)	32 (29.4)	13 (21.3)	.254	14 (24.1)	13 (22.4)	.826
ASA score (≥3), n (%)	9 (8.3)	6 (9.8)	.728	3 (5.2)	6 (10.3)	.298
RENAL score, median (IQR)	8 (7‐9)	8 (7‐9)	.535	8 (7‐9)	8 (7‐9)	.635
Left tumor, n (%)	49 (45.0)	32 (52.5)	.347	29 (50.0)	32 (55.2)	.577
Solitary kidney, n (%)	14 (12.8)	2 (3.3)	**.040**	3 (5.2)	2 (3.4)	.648
Hilar tumor, n (%)	21 (19.3)	16 (26.2)	.291	12 (20.7)	15 (25.9)	.510
Mean tumor size (cm), mean (SD)	4.9 (0.7)	5.0 (0.7)	.475	4.9 (0.7)	5.0 (0.7)	.327
Preoperative creatinine, umol/L, mean (SD)	90.1 (25.3)	87.1 (19.4)	.428	85.9 (24.5)	87.0 (19.6)	.788
Preoperative eGFR, mL/min, mean (SD)	84.2 (18.7)	88.5 (14.2)	.098	88.5 (17.0)	88.7 (14.4)	.950
Preoperative CKD, n (%)	12 (11.0)	1 (1.6)	**.027**	1 (1.7)	1 (1.7)	1.000
Preoperative hemoglobin, g/L, mean (SD)	116.3 (20.6)	123.0 (17.2)	**.025**	122.7 (17.6)	121.7 (15.8)	.750

Bold indicates statistical significance value (*P* < 0.05)

Abbreviations: ASA: American Society of Anesthesiologists; CKD: chronic kidney disease; IQR: inter‐quartile range; SD: standard deviation.

Perioperative outcomes after PSM have been exhibited in Table 2. The proportion of retroperitoneal approach did not differ significantly between the two groups (58.6% vs 65.5%, *P* = .444). The LPN group was in connection with a significantly longer mean OT (219.1 vs 198.8 minutes, *P* = .040), longer mean WIT (24.2 vs 22.4 minutes, *P* = .023), and longer median postoperative hospital stay length (9 vs 7.5 days, *P* = .023). There were no significant differences in the rates of postoperative complication, conversion to radical or open surgery, and PM between the LPN and RPN groups. The median EBL was also comparable between the two groups (200 vs 160 mL, *P* = .405). The statistical significances for all pathological outcomes, namely, pathologic stage, fuhrman grade, and histologic subtype, were calculated with a two‐sided *P* > .05 after the PSM (Table 2). Renal functional results have been revealed in Table 2. The comparability of mean eGFR between the two groups remains existing within postoperative 1 week (73.2 vs 73.9 mL/min, *P* = .805) and at the last follow‐up (77.2 vs 80.8 mL/min, *P* = .293), and the eGFR decrease in baseline remains different insignificantly within postoperative 1 week (−15.4 vs −14.8 mL/min, *P* = .358) and at the last follow‐up (−11.3 vs −7.9 mL/min, *P* = .077). The de novo CKD at last follow‐up occurred in a higher proportion in the LPN group in spite of the statistical insignificance (19.0% vs 6.9%, *P* = .053).

**Table 2:** Perioperative outcomes for LPN and RPN after propensity score matching

	LPN (n = 58)	RPN (n = 58)	*P* value
Surgical approach (retroperitoneal), n (%)	34 (58.6)	38 (65.5)	.444
Operating time, min, mean (SD)	219.1 (33.0)	198.8 (65.9)	**.040**
Estimated blood loss, mL, median (IQR)	200 (100‐300)	160 (100‐212.5)	.405
Warm ischemia time, min, mean (SD)	24.2 (4.5)	22.4 (3.6)	**.023**
Transfusion, n (%)	11 (19.0)	5 (8.6)	.106
Conversion, n (%)	5 (8.6)	2 (3.4)	.242
To open surgery	2 (3.4)	0	.496
To radical surgery	4 (6.9)	2 (3.4)	.402
Postoperative hospital stay, days, median (IQR)	9 (7‐10.5)	7.5 (6‐9)	**.023**
Postoperative complications, n (%)	17 (29.3)	12 (20.7)	.284
Low grade (Clavien‐Dindo I–II), n (%)	13 (22.4)	9 (15.5)	.343
High grade (Clavien‐Dindo III–IV), n (%)	4 (6.9)	3 (5.2)	.697
Pathologic stage			.701
pT1b, n (%)	39 (67.2)	44 (75.9)	
pT2, n (%)	2 (3.4)	1 (1.7)	
pT3a, n (%)	5 (8.6)	4 (6.9)	
Fuhrman grade			.741
Low (≤2), n (%)	31 (53.4)	34 (58.6)	
High (≥3), n (%)	7 (12.1)	5 (8.6)	
Not specified, n (%)	8 (13.8)	10 (17.2)	
Histologic subtype			.469
Benign, n (%)	12 (20.7)	9 (15.5)	
Malignant, n (%)	46 (79.3)	49 (84.5)	
Positive surgical margin, n (%)	1 (1.7)	2 (3.4)	1.000
eGFR within postoperative 1 wk, mL/min, mean (SD)	73.2 (18.0)	73.9 (15.4)	.805
eGFR decrease in baseline, mL/min, mean (SD)	−15.4 (3.4)	−14.8 (3.5)	.358
Last follow‐up eGFR, mL/min, mean (SD)	77.2 (21.1)	80.8 (14.9)	.293
eGFR decrease in baseline, mL/min, mean (SD)	−11.3 (13.0)	−7.9 (6.3)	.077
De novo CKD at last follow‐up, n (%)	11 (19.0)	4 (6.9)	.053

Bold indicates statistical significance value (*P* < 0.05)

Abbreviations: CKD: chronic kidney disease; IQR: inter‐quartile range; SD: standard deviation.

**Figure 1:**
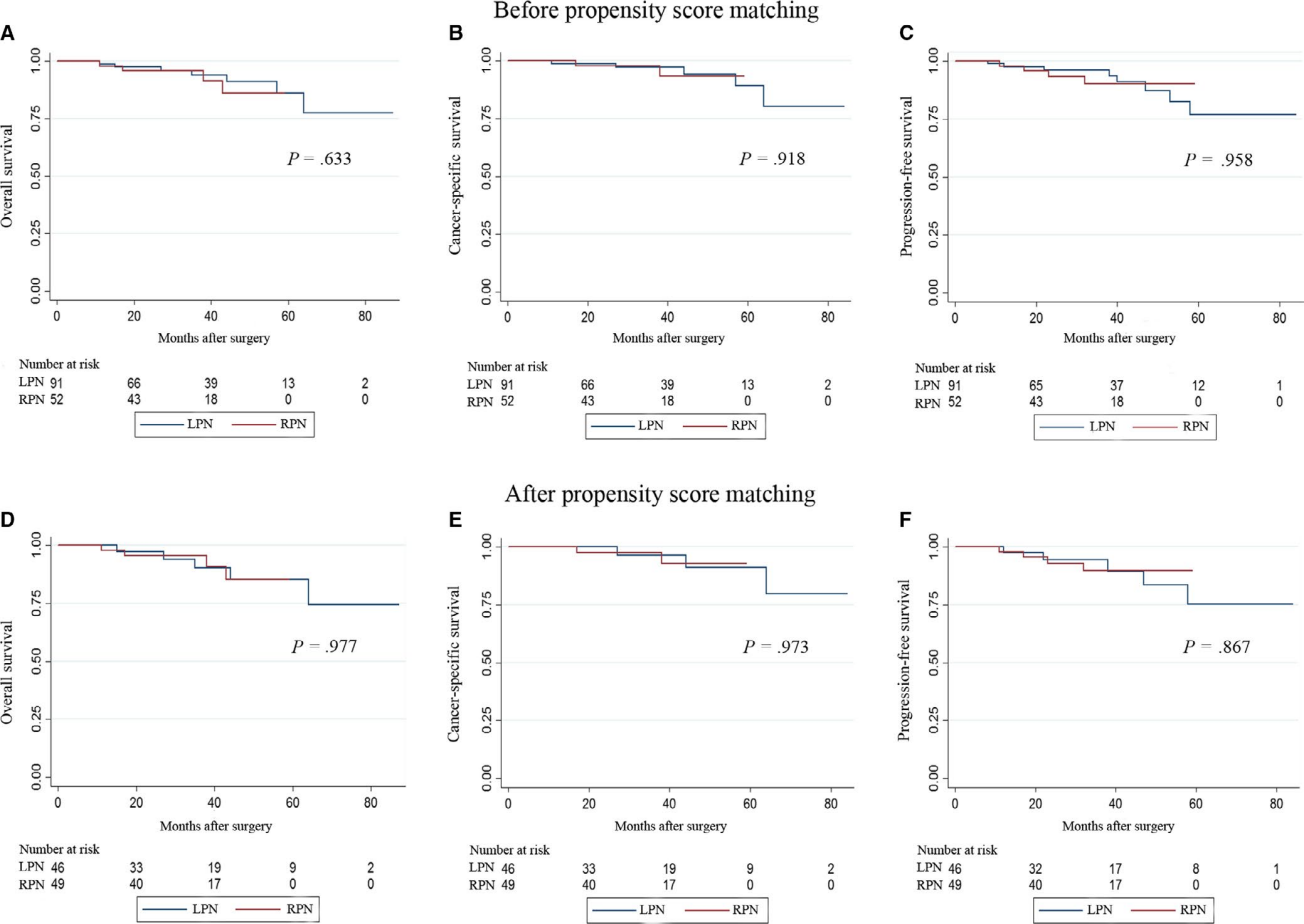
Kaplan‐Meier analyses for overall, cancer‐specific and progression‐free survivals according to the surgical type. A, Overall survivals of LPN and RPN before propensity score matching. B, Cancer‐specific survivals of LPN and RPN before propensity score matching. C, Progression‐free survivals of LPN and RPN before propensity score matching. D, Overall survivals of LPN and RPN after propensity score matching. E, Cancer‐specific survivals of LPN and RPN after propensity score matching. F, Progression‐free survivals of LPN and RPN after propensity score matching

**Figure 2:**
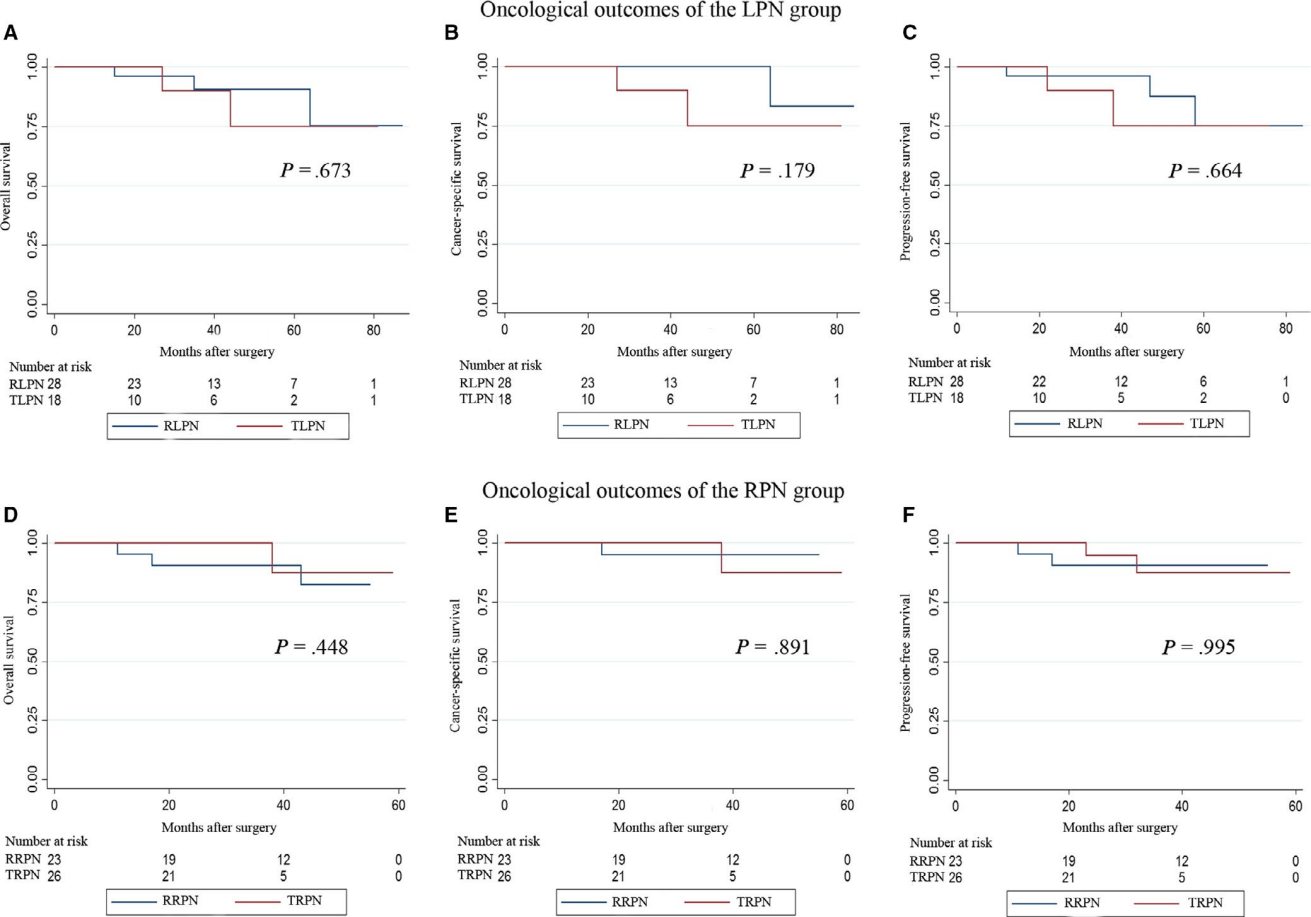
Kaplan‐Meier analyses for overall, cancer‐specific and progression‐free survivals of the LPN and RPN groups according to the surgical approaches. A, Overall survivals of TLPN and RLPN after propensity score matching. B, Cancer‐specific survivals of TLPN and RLPN after propensity score matching. C, Progression‐free survivals of TLPN and RLPN after propensity score matching. D, Overall survivals of TRPN and RRPN after propensity score matching. E, Cancer‐specific survivals of TRPN and RRPN after propensity score matching. F, Progression‐free survivals of TRPN and RRPN after propensity score matching. RLPN: retroperitoneal LPN; TLPN: transperitoneal LPN; RRPN: retroperitoneal RPN; TRPN: transperitoneal RPN

The median follow‐up periods of the LPN and RPN groups were 39.5 and 31.0 months, respectively, before the PSM (*P* = .315). Within the entire cohort, the occurrences of overall death, cancer‐specific death and tumor progression have happened to 7 and 4 patients, 5 and 1 patient, 10 and 4 patients in the LPN and RPN groups, respectively, during the follow‐up intervals. No significant differences in OS, CSS, and PFS between the two groups were detected utilizing the Kaplan‐Meier analysis (Figure 1A‐C). After the PSM, the median follow‐up for the LPN and RPN groups was 36.0 and 31.0 months, respectively (*P* = .414). The incidences of overall death, cancer‐specific death and tumor progression have occurred to 5 and 4 patients, 3 and 1 patient, 5 and 3 patients, respectively, in the LPN and RPN groups. The statistical similarity in OS, CSS and PFS remained after the PSM between the two groups (Figure 1D‐F). When we stratified the patients in the LPN and RPN groups into two subgroups according to the surgical approaches, no significant differences were found in surgical approaches in both the LPN and RPN groups (Figure 2). With respect to those with benign tumors, no occurrences of death or tumor progression have happened in the LPN and RPN groups during the 37‐month and 32‐month median follow‐up periods, respectively.

## 4 DISCUSSION

As the most distinctive and attractive advantage of PN over RN, better renal functional preservation would ultimately translate into the benefit of better overall survival,^2^, ^19^ thus resulting in a strong recommendation of PN for localized T1a and even T1b tumors in spite of surgical approach.^1^ The constantly improved surgical technique and better understanding of biologic characteristics of renal tumor have led to the prosperity of minimally invasive techniques.^20^ LPN, a feasible and effective treatment for selected endophytic hilar tumors in highly experienced hands,^21^, ^22^ could be more challenging when treating more complex renal tumors. Due to the main superiority of robotic surgery, RPN was considered as a widely accepted alternative to open PN and LPN when surgically treating renal tumors.^9^ Tumor complexity, one of the important determinants of surgical and oncological outcomes,^9^ deeply impacts the decision of surgical approach.^23^ Several studies have demonstrated the safety and feasibility of minimally invasive PN for complex renal tumor,^9^, ^10^, ^11^, ^24^, ^25^ especially for complex T1a renal tumors.^26^ RPN has been considered as a feasible option in management of ≥4 cm renal tumors with moderate or high nephrometry scores as well.^25^ The controversy about the advantage of LPN and RPN for anatomically complex T1b renal tumors with a RENAL nephrometry score ≥7 remains unknown and debated.^14^

The present study was the first analysis focusing on the perioperative, renal functional, and oncological outcomes of LPN and RPN approaching anatomically complex T1b renal tumors with a RENAL nephrometry score ≥7. An enhanced balance about preoperative characteristics was achieved to reduce the impact of potential selection bias and confounders utilizing the PSM method. After the PSM method, RPN has an advantage over LPN in shortening OT, WIT, and postoperative hospital stay length when managing anatomically complex T1b renal tumors.

Intracorporeal suturing within limited WIT is technically challenged under restricted movement of laparoscopic forceps.^5^, ^27^ All these difficulties have been largely settled by surgical robot with the enhanced dexterity, better visualization, and tremor filtration.^11^ All these improvements in technique and equipment have resulted in a shorter OT, a reduction of WIT, and a decline of postoperative hospital stay length in our analysis, which has been proven in previously published studies comparing RPN and LPN.^11^, ^28^, ^29^, ^30^ The perioperative outcomes vary subtly from different studies^11^, ^30^, ^31^, ^32^ comparing RPN and LPN for complex renal tumor probably due to the diverse surgeon experience. Leow et al^33^ found that RPN was in association with reducing WIT compared with LPN regardless of whether the center was high or low volume, illustrating the superiority of the robotic approach in preventing nephron ischemia. In their subgroup analysis including three studies^30^, ^31^, ^32^ focusing on complex renal tumors, a significantly shorter WIT (*P* = .04), OT (*P* = .011) and postoperative hospital length (*P* = .029) were found in the RPN group compared with that in the LPN group in spite of the comparable EBL (*P* = .961),^33^ which is consistent with our results. This consistency highlights the unique superiority of RPN in managing these complex tumors.

The proportion of overall complications after RPN ranges from 9% to 33.3%, while the overall complication rate after LPN varies from 5% to 33%.^34^, ^35^ Most of these complications were in Clavien‐Dindo ≤2, namely in low grade. The lowest ratio of overall complications after RPN for complex renal tumors was 20.2%.^9^, ^10^, ^12^, ^30^, ^31^ The results about overall complications in the presented analysis were similar to these in reported studies. A reduction of postoperative complications including the low and high grade ones, as an advantage of RPN over LPN,^27^, ^33^ seems disappeared when surgically managing complex renal tumors.^11^, ^33^ The differences about the rates of conversion and transfusion were also insignificant in previous study^11^ and our analysis. The presented analysis illustrated the equivalence in the safety of LPN and RPN for anatomically complex T1b renal tumors.

The quality and quantity of preserved nephron were in strong association with renal function recovery after PN.^36^ The mean tumor size was similar in the LPN and RPN groups in the presented study, probably resulting in comparable resection volume, and the mean WIT in both groups were less than 25 minutes, which was proposed as the watershed of achieving trifecta.^37^ No evidence supported that limited ischemia time (≤25 minutes) has a higher risk of reducing renal function after PN compared to a “zero ischemia” technique.^38^ Gu et al^39^ observed significant differences in early eGFR change, which could be explained by the temporary effect of renal artery clamp time. But the impact of WIT on late renal function after RPN might be limited.^40^ Choi et al^41^ also found that the stationary overall eGFR after LPN or RPN may be functionally compensated by the contralateral healthy kidney unless WIT exceeds 28 minutes. Leow et al^33^ concluded that the postoperative eGFR change was similar between the LPN and RPN groups in an updated meta‐analysis of 4919 patients. Long et al^30^ also found that surgical approach was not a predictor of postoperative eGFR or postoperative percentage change in eGFR when approaching complex renal tumors. Our results exhibited the similarity in renal function preservation after LPN or RPN for anatomically complex T1b renal tumors both within postoperative 1 week and at last follow‐up, which could be attributed to the limited WIT and similar mean tumor size in the LPN and RPN groups, despite of the superiority of robotic surgery in precision in preserving functional nephrons.

Oncological control is of original significance when choosing the approach to surgically treat renal tumors.^11^ The median follow‐up lengths of both groups in our study were longer than those reported in previous analyses^11^, ^30^, ^31^, ^32^ for complex renal tumors. The inadequate follow‐up time had also precluded Gu et al^39^ from drawing any conclusions in oncological outcomes of patients with >4 cm renal tumors after LPN and RPN. In our analysis, the insignificant differences in OS, CSS, and PFS between the two groups remain stable before and after matching the preoperative features. The similar pathologic characteristics and rate of PM after PSM enhance the strength of oncological outcomes. Kizilay et al^42^ found that operation method was not the predictive factor for 5‐year CSS. The PM rates were comparable even at low‐volume centers, emphasizing the oncologic equivalence of RPN to LPN.^33^ The similarity in the rate of PM between the two surgical approaches was presented by Choi et al^5^ after pooling data from 11 different centers. After the PSM, the comparability of the rate of CKD stage before and after surgery may help to understand the similar CSS in the two groups, in spite of the different clinical impacts on CSS of the medically and surgically induced CKD.^43^ In spite of different surgical challenges faced when employing retroperitoneal or transperitoneal approach, many studies have reported the similar oncological outcomes for these two approaches,^1^, ^44^, ^45^ which was confirmed again in our subgroup analyses.

Several limitations were unavoidable in the present study. Firstly, potential selection bias and confounders may exist out of control regardless of matching all preoperative characteristics in this retrospectively designed study. Secondly, the structural weakness in acquiring and gathering the data may be unavoidable in retrospective study. Certain postoperative complications especially the minor ones may be underestimated even though all medical records were scrutinized exhaustively. Moreover, all RPN procedures were completed during the latter study period due to the developmental features of the surgical techniques. All surgeries were performed in extensively experienced hands. Finally, the survival outcomes may be influenced by the diverse salvage or adjuvant therapies in patients with disease progression.

Despite these shortcomings, our analysis is the first one concentrating on the safety and efficiency of RPN and RPN for anatomically complex T1b renal tumors with a RENAL nephrometry score ≥7. Underlying selection bias and confounders were restricted utilizing the PSM method to enhance the strength of our conclusions.

## 5 CONCLUSIONS

For patients with anatomically complex T1b renal tumors with a RENAL nephrometry score ≥7, RPN had an advantage over LPN in reducing the OT, WIT, and postoperative hospital stay length without increasing the risk of complications and weakening the oncological control, while the two surgical methods were similar in renal functional preservation. Our present conclusions need to be further validated in prospectively randomized studies with large samples and long‐term follow‐up period.

## CONFLICT OF INTEREST

The authors declare that they have no competing interests.

## Data Availability

The data that support the findings of this study are available from the corresponding author upon reasonable request.
